# Association Between Food Insecurity and Metabolic Dysfunction-Associated Steatotic Liver Disease: A Cross-Sectional Study in South Korea

**DOI:** 10.3390/nu18172806

**Published:** 2026-08-27

**Authors:** Seong-Uk Baek, Jin-Ha Yoon

**Affiliations:** 1 Graduate School, Yonsei University College of Medicine, Seoul 03722, Republic of Korea; 2 Department of Preventive Medicine, Yonsei University College of Medicine, Seoul 03722, Republic of Korea; 3 Institute for Innovation in Digital Healthcare, Yonsei University Health System, Seoul 03722, Republic of Korea; 4 The Institute for Occupational Health, Yonsei University College of Medicine, Seoul 03722, Republic of Korea

**Keywords:** metabolic dysfunction, metabolic syndrome, food security, fatty liver disease

## Abstract

**Background/Objectives:** Metabolic dysfunction-associated steatotic liver disease (MASLD) has become an important public health burden. However, the association of food insecurity with MASLD remains understudied. Accordingly, this study analyzed the association between food insecurity and MASLD in Korean adults. **Methods:** We used data from 12,697 adults drawn from a nationally representative survey. Food insecurity was determined using the Household Food Security Survey Module. Food insecurity was categorized into no, mild, and moderate-to-severe. MASLD was determined by a hepatic steatosis index >36 in conjunction with at least one metabolic abnormality. The association was analyzed using logistic regression. **Results:** Among the participants, 95.5%, 3.7%, and 0.8% were classified as having no, mild, and moderate-to-severe food insecurity, respectively. Compared to no food insecurity, the odds ratios (ORs) (95% CIs [confidence intervals]) for MASLD were 1.40 (1.09–1.81) for mild food insecurity and 1.76 (1.06–2.90) for moderate-to-severe food insecurity. Additionally, the continuous HFSSM score was directly associated with MASLD (OR: 1.07; 95% CI: 1.02–1.12). **Conclusions:** This nationally representative study showed that, compared with no food insecurity, mild and moderate-to-severe food insecurity were associated with MASLD.

## 1. Introduction

Metabolic dysfunction-associated steatotic liver disease (MASLD) is a considerable population health issue [1]. Formerly termed non-alcoholic fatty liver disease (NAFLD), it was redefined to reflect the need for a positive definition that recognizes the role of metabolic abnormalities in its pathophysiology [2]. In South Korea, the prevalence of MASLD was estimated to be 31.0% in 2022–2023, a notable increase from 25.0% in 2007–2009 [3]. This trend highlights the urgent need to implement effective preventive strategies to reduce the health burden of MASLD [4].

Food insecurity is defined as a condition in which individuals are unable to access safe and nutritious food to maintain health [5]. It has been estimated that approximately 28.0% of the global population was exposed to food insecurity in 2024, with substantial disparities across regions and countries according to socioeconomic status [6]. As a high-income country in Asia, South Korea has a relatively low prevalence of food insecurity, with approximately 4.0% of its population experiencing food insecurity [7,8].

Food insecurity has been linked to poor health consequences, including sarcopenia [9], cardiovascular disease [10], and mental disorders [11]. Additionally, food insecurity has been related to metabolic abnormalities, including obesity [12], low high-density lipoprotein cholesterol (HDL-C) [13], and hypertension [14]. Among adults in the US, those exposed to food insecurity had an increased prevalence of metabolic syndrome compared to those without food insecurity [15]. Although the precise mechanisms explaining the connection between food insecurity and metabolic abnormalities remain poorly understood, those exposed to food insecurity often have poorer dietary quality, characterized by greater consumption of energy-dense, nutrient-poor foods, including foods with added sugars, confectionery, and processed meats [16,17], potentially leading to metabolic abnormalities.

Although food insecurity has been linked to metabolic abnormalities, few studies have investigated its association with MASLD. An ecological study across 204 countries revealed that food insecurity prevalence is positively correlated with the MASLD prevalence in high-income countries [18]. Prior studies also found that those with food insecurity had a higher likelihood of having MASLD than those without food insecurity [19,20]. Similarly, one study found that food insecurity was associated with MASLD in adolescents [21]. However, the existing literature has predominantly focused on populations in the US [19,20,21], potentially restricting the generalizability of their results to other regions. Given the substantial differences in food security, dietary practices, and MASLD prevalence across geographic regions and populations, further research in diverse populations is warranted. Additionally, the association of food insecurity with MASLD has not been investigated in South Korea and a broader Asian population. To address this knowledge gap, we examined the association between food insecurity and MASLD among South Korean adults.

## 2. Methods

### 2.1. Study Population

**Figure 1:**
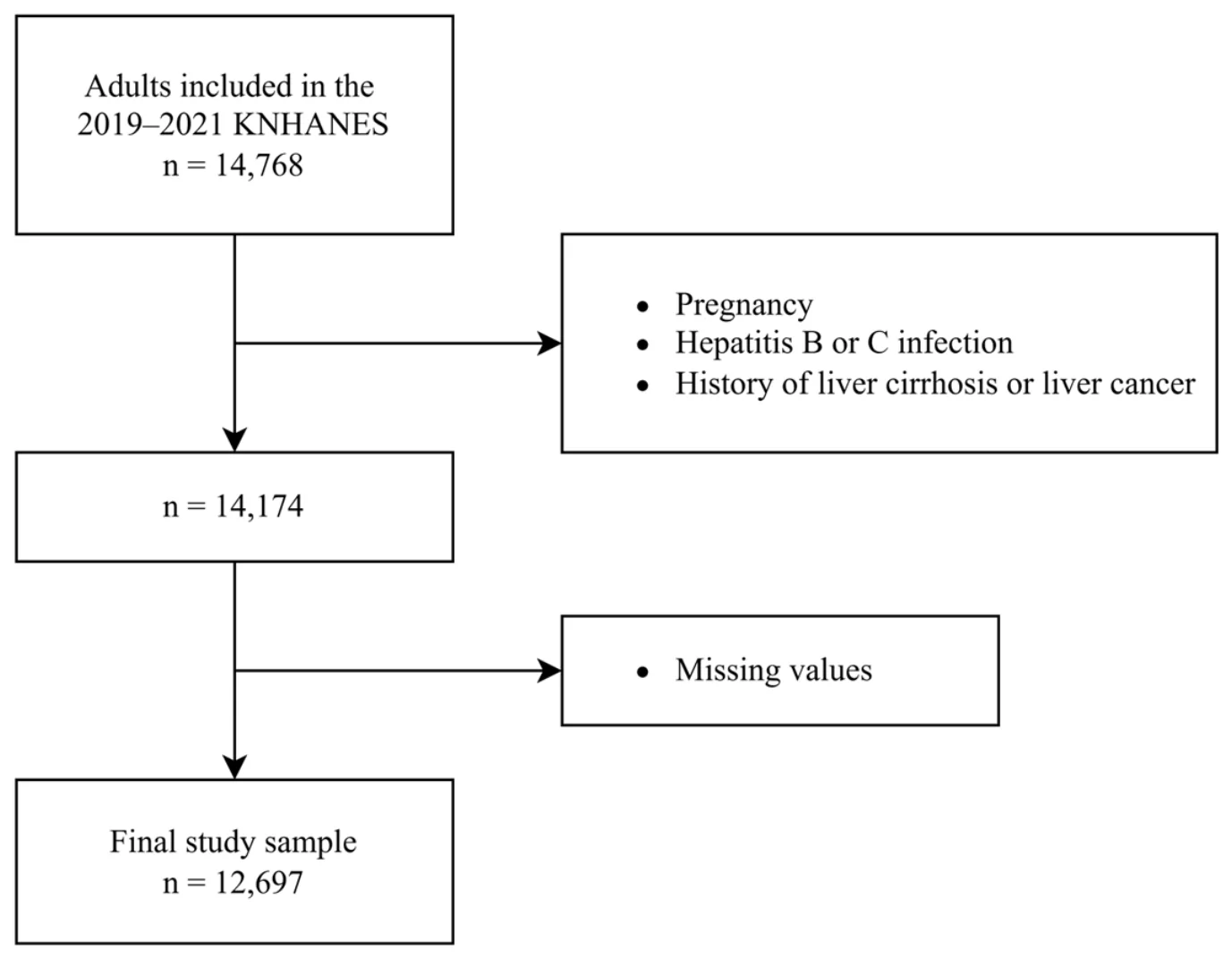
Study sample selection process.

We analyzed participants from the 2019–2021 Korea National Health and Nutrition Examination Survey (KNHANES), the years in which food insecurity was measured. The KNHANES is a representative survey of the South Korean population that uses multistage clustered sampling [22]. Figure 1 shows a diagram of the sample selection process. Among the 14,768 participants aged >18 years included in the survey, those who were pregnant or had hepatitis B virus or hepatitis C virus infection, liver cirrhosis, a history of liver cancer, or missing data on the study variables were excluded. Ultimately, 12,697 adults were analyzed in this study. Written informed consent was provided by all study participants. The study was approved by the Institutional Review Boards of the Korea Disease Control and Prevention Agency (2018-01-03-C-A; 2018-01-03-2C-A; 2018-01-03-5C-A).

### 2.2. Food Insecurity

Food insecurity was determined by the 18-item Household Food Security Survey Module (HFSSM) [23]. The Korean version of the HFSSM has demonstrated good reliability and validity for evaluating food insecurity [24]. The HFSSM consists of items assessing difficulties experienced in accessing adequate food during the previous 12 months. Each item was scored as 0 or 1. Ten items were administered to all participants, whereas the remaining eight were administered only to individuals from households with children. Detailed information on the questionnaire has been reported previously [24]. The total score ranges from 0 to 10 for those from households without children and from 0 to 18 for those from households with one or more children, with higher scores indicating greater food insecurity. Following the cutoff values recommended by the guideline [23], food insecurity was classified into three categories. For those from households with one or more children, scores of 0–2, 3–7, and 8–18 represented no, mild, and moderate-to-severe food insecurity, respectively. Conversely, for those from households without children, the corresponding ranges were defined as 0–2, 3–5, and 6–10. The detailed survey items are listed in Table S1.

### 2.3. MASLD

MASLD was determined by the presence of hepatic steatosis and one or more metabolic abnormalities, according to the current guideline [2]. Hepatic steatosis was determined based on the hepatic steatosis index (HSI), a validated index developed in South Korea [25]. Prior studies have demonstrated that the HSI has reasonable diagnostic performance for detecting MASLD [26,27,28,29]. The HSI was defined as 8 × (ALT/AST ratio) + BMI (+2 if diabetes mellitus and +2 if female), where ALT and AST denote serum alanine aminotransferase and aspartate aminotransferase levels, respectively. Following the methodology used in the literature [25,30], an HSI >36 was considered indicative of hepatic steatosis. We chose an HSI cutoff of >36, which is characterized by high specificity and relatively low sensitivity [25]. This cutoff may reduce false-positive classification and provide a more stringent identification of MASLD cases.

Metabolic abnormalities were identified based on the presence of one or more of the following cardiometabolic risk factors [2,31]: (i) BMI ≥ 23 kg/m^2^ or waist circumference ≥ 90 cm in men or ≥85 cm in women; (ii) fasting plasma glucose ≥ 100 mg/dL, HbA1c ≥ 5.7%, or use of insulin or oral hypoglycemic agents; (iii) BP ≥ 130/85 mmHg or use of antihypertensive medications; (iv) TG levels ≥ 150 mg/dL or use of lipid-lowering medications; and (v) HDL-C < 40 mg/dL in males or <50 mg/dL in females or use of lipid-lowering medications.

### 2.4. Control Variables

The analyses encompassed the following factors: sex, age, region (urban or rural), income (Q1–Q4), education (middle school or lower, high school, college or higher), marital status (married or unmarried), smoking status (no or yes), physical activity (no or yes), and alcohol use. Income status was grouped into quartiles based on the average monthly income. Physical activity was determined using the Global Physical Activity Questionnaire [32] and classified according to whether individuals participated in ≥150 min/wk of physical activity, following the World Health Organization guidelines [33]. Finally, alcohol use status was measured using the Alcohol Use Disorders Identification Test–Consumption, which was validated in the Korean population [34].

Dietary quality was assessed using the Korean Healthy Eating Index (KHEI) [35,36]. The KHEI comprises 14 components, including adequacy components (adequate intake of protein-rich foods, vegetables, fruits, and dairy products), moderation components (limited intake of saturated fatty acids, sodium, and sugars), and balance components (balanced energy intake). The total KHEI score ranges from 0 to 100, with higher scores indicating better dietary quality. Detailed information on the KHEI components and its validation has been described elsewhere [35,36].

### 2.5. Quality Control

To ensure the quality of questionnaire data and biological sample collection, the KNHANES implements standardized quality control procedures [37]. Interviewers undergo periodic training and are regularly evaluated by experts to maintain competency and receive performance feedback. Blood samples are collected by certified medical laboratory technologists or registered nurses following standardized protocols, with regular quality assurance procedures applied throughout sample collection, handling, storage, and processing. In addition, all laboratory analyses are conducted in government-certified laboratories, where standardized analytical procedures are used to ensure the precision and accuracy of all laboratory measurements.

### 2.6. Data Analysis

The sample characteristics were summarized according to the food insecurity status. The association of food insecurity with MASLD was examined using logistic regressions. Odds ratios (ORs) and 95% confidence intervals (CIs) were estimated. Two models were sequentially fitted. Model 1 was adjusted for sex, age, region, household income, educational attainment, marital status, smoking status, physical activity, and alcohol use. Model 2 was additionally adjusted for dietary quality, as measured by the KHEI. Considering that dietary quality may represent an intermediate variable in the association between food insecurity and MASLD, Model 1 estimates the total association between food insecurity and MASLD, whereas Model 2 estimates the direct association independent of dietary quality. Subsequently, the association between food insecurity and each metabolic abnormality was analyzed. Data were analyzed using R (version 4.6.0). The complex survey design was accounted for in the logistic regressions using the “survey” package. Following the KNHANES analysis guidelines, the annual sampling weights were divided by the number of pooled survey years (i.e., three) to derive the final weights for the pooled dataset.

Sensitivity analyses were performed. First, multiple imputation was used to handle missing data. A total of 20 complete datasets were imputed using the “mice” package, and the estimates were pooled [38]. Second, alternative indices for identifying hepatic steatosis were employed, including the Framingham Steatosis Index (FSI) [39] and the Zhejiang University (ZJU) index [40]. The detailed scoring systems are presented in Table S2.

## 3. Results

As Table 1 shows, 95.5%, 3.7%, and 0.8% were classified as having no, mild, and moderate-to-severe food insecurity, respectively. The overall sample comprised 50.0% men and 50.0% women, with a mean (standard deviation) age of 47.4 (16.6) years. Overall, 63.7% of participants were married, 18.7% were current smokers, and 45.8% met the recommended level of physical activity. In comparison with participants with no food insecurity, those having food insecurity tended to be older, reside in rural areas, have lower income and education levels, be unmarried, and be smokers.

Table 2 shows the dietary quality across food insecurity groups. The overall mean (standard deviation) KHEI was 58.0 (13.3) in participants with no food insecurity, 57.5 (13.4) in individuals with mild food insecurity, and 50.3 (15.0) in individuals with moderate-to-severe food insecurity. Compared with the non-food insecurity group, the moderate-to-severe food insecurity group had lower adequacy and balance scores but a higher moderation score.

**Figure 2:**
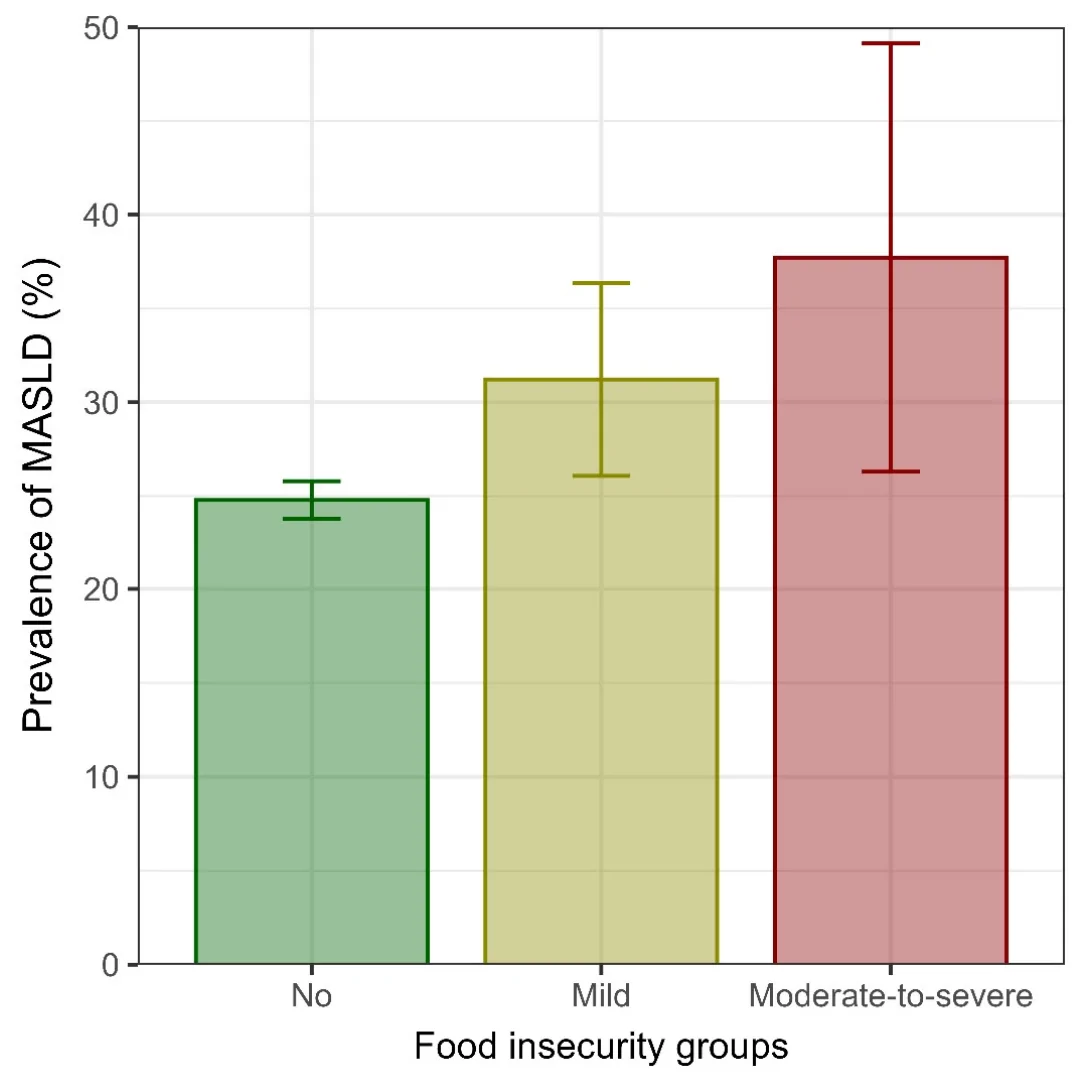
Metabolic dysfunction-associated steatotic liver disease (MASLD) prevalence by food insecurity groups. Survey weights were applied.

The overall prevalence of MASLD was 25.1% (N = 3075). The prevalence of MASLD across the food insecurity groups is shown in Figure 2. The prevalence of MASLD was 24.8% in participants with no food insecurity, 31.2% in individuals with mild food insecurity, and 37.7% in individuals with moderate-to-severe food insecurity.

**Figure 3:**
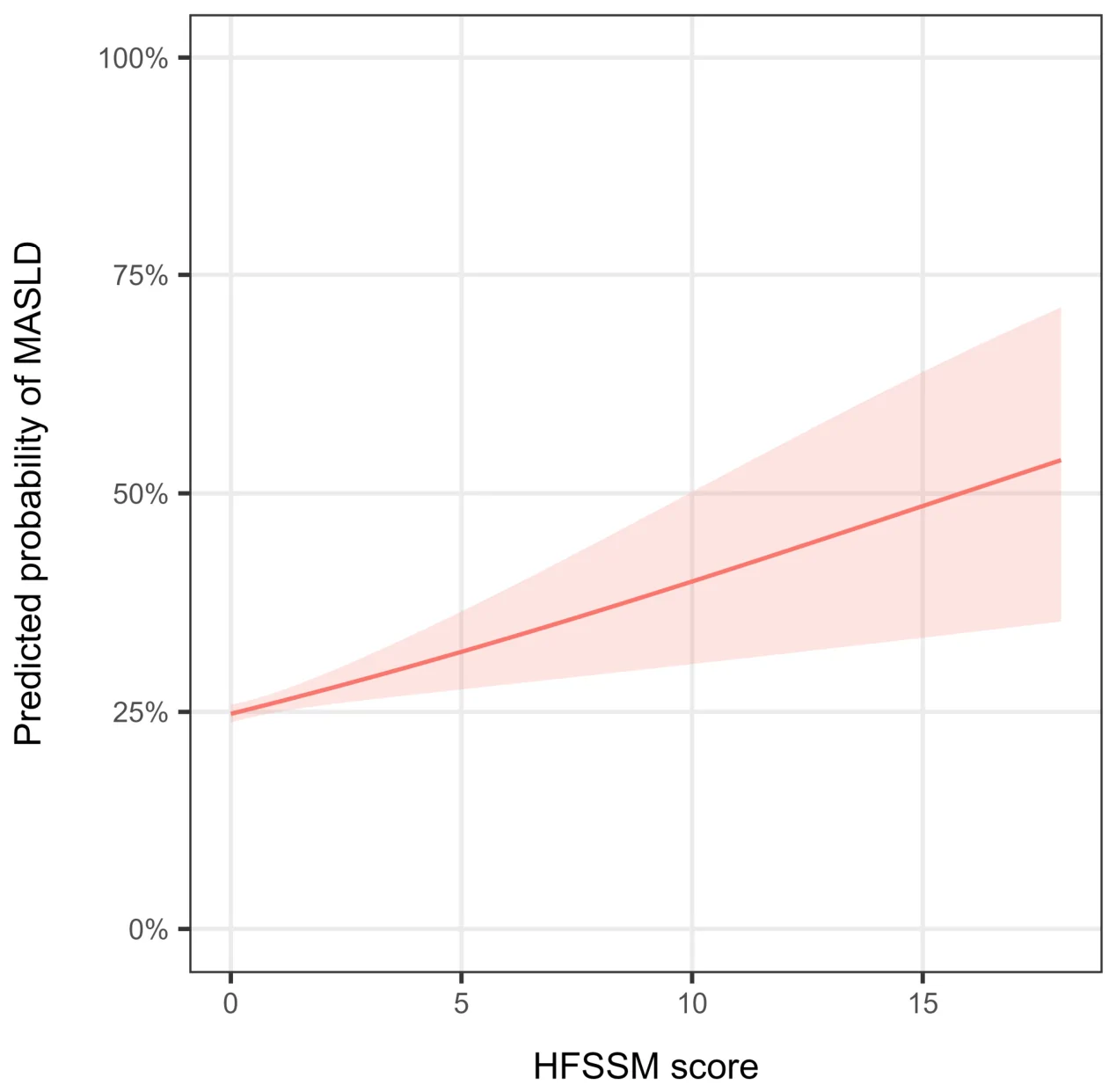
Predicted probability of metabolic dysfunction-associated steatotic liver disease (MASLD) according to the continuous Household Food Security Survey Module (HFSSM) score.

Table 3 shows the association of food insecurity with MASLD in the logistic regression models. In Model 1, using participants with no food insecurity as the reference group, the adjusted ORs (95% CIs) for MASLD were 1.42 (1.10–1.83) for mild food insecurity and 1.83 (1.10–3.02) for moderate-to-severe food insecurity. When analyzed as a continuous variable, the HFSSM score was positively associated with MASLD (OR: 1.07; 95% CI: 1.03–1.12). In the model additionally adjusting for the KHEI score (Model 2), the associations slightly attenuated; the adjusted ORs (95% CIs) for MASLD were 1.40 (1.09–1.81) for mild food insecurity and 1.76 (1.06–2.90) for moderate-to-severe food insecurity. When analyzed as a continuous variable, the HFSSM score was positively associated with MASLD (OR: 1.07; 95% CI: 1.02–1.12). Figure 3 illustrates the predicted probability of MASLD based on a model incorporating the continuous HFSSM score in the fully adjusted logistic model (Model 2).

Table 4 shows the association of food insecurity with metabolic abnormalities. In the fully adjusted models (Model 2), compared with the non-food insecurity group, moderate-to-severe food insecurity was associated with elevated BP (OR, 1.84; 95% CI, 1.08–3.13) and reduced HDL-C (OR, 2.11; 95% CI, 1.22–3.62). Additionally, moderate-to-severe food insecurity was marginally associated with hypertriglyceridemia (OR, 1.65; 95% CI, 0.99–2.74).

Table 5 shows the association of a continuous HFSSM score with metabolic abnormalities according to subgroups by sex, age, obesity, and diabetes mellitus. The association between the HFSSM score and MASLD was more pronounced among women and those with obesity.

Using multiple imputation (Table S3, the adjusted ORs (95% CIs) for MASLD were 1.39 (1.10–1.76) for mild food insecurity and 1.68 (1.07–2.63) for moderate-to-severe food insecurity. Using FSI (Table S4), the corresponding adjusted ORs (95% CIs) were 1.36 (1.06–1.76) and 2.19 (1.31–3.68), respectively. Similarly, using the ZJU index, the corresponding adjusted ORs (95% CIs) were 1.29 (1.01–1.66) and 1.75 (1.04–2.93), respectively. Overall, the positive association of both mild and moderate-to-severe food insecurity with MASLD remained in the sensitivity analyses.

## 4. Discussion

This nationally representative cross-sectional study analyzed the association of food insecurity with MASLD among adults in South Korea. In comparison with participants without food insecurity, individuals exposed to mild or moderate-to-severe food insecurity had higher odds of having MASLD after adjustment for socio-demographic characteristics. Considering the relatively modest strength of the association, as reflected by the ORs, and the low prevalence of moderate-to-severe food insecurity in the population, the clinical and public health relevance of these findings may be limited. However, our findings provide insights into the potential association between food insecurity and MASLD and highlight the need for further prospective research to elucidate the temporal relationship between food insecurity and MASLD.

The findings of this study indicated that approximately 4.3% of the Korean population experienced food insecurity during the study period (2019–2021). This prevalence is lower than the global estimate of food insecurity and is comparable to that reported in other high-income countries, such as Japan and several European countries [6]. Multiple socioeconomic factors, including relatively high income levels and well-established social welfare systems designed to improve food security among low-income populations, may have contributed to this comparatively low prevalence [41].

In the present study, the prevalence of MASLD among Korean adults was 25.1%. By comparison, a previous meta-analysis estimated that approximately 38% of adults worldwide are affected by MASLD [42]. Nevertheless, the prevalence of MASLD has been increasing both in Korea and globally [3]. It has been hypothesized that this trend may be attributed to the adoption of Western dietary patterns, increasingly sedentary lifestyles, and the growing burden of metabolic dysfunction [3]. Additionally, studies have shown that exposure to poor working environments or environmental exposure may be associated with metabolic abnormalities or steatotic liver disease [43,44,45,46,47]. Our findings indicate that approximately one in four Korean adults have MASLD, underscoring the need for effective public health policies and preventive strategies to improve the prevention and management of MASLD and mitigate its associated health burden.

The present findings corroborate those of prior studies that have reported an association between food insecurity and MASLD or NAFLD. An ecological study across 204 countries reported a positive correlation between food insecurity and MASLD prevalence in high-income countries [18]. Prior cross-sectional studies have also reported an association between food insecurity and MASLD among adolescents and adults in the United States [19,20,21]. Similarly, earlier studies have revealed that food insecurity is associated with NAFLD [48,49]. Additionally, studies have linked food insecurity to cardiometabolic risk factors and metabolic abnormalities [7,12,50,51]. While the prevalence of food insecurity was lower than that reported in the US, the association between food insecurity and MASLD was similar to that observed in previous studies from the US [19,20]. Although South Korea has broader coverage of social protection systems, including health insurance and food security policies [52], the observed significant association between food insecurity and MASLD suggests that this association may persist even in settings with relatively comprehensive social safety nets.

Although the precise mechanisms underlying the association between food insecurity and MASLD remain unclear, one plausible pathway involves poor diet quality and unhealthy dietary behaviors [53,54]. Owing to financial constraints, individuals exposed to food insecurity tend to consume fewer fruits, vegetables, and dairy products [55] and are more likely to consume ultra-processed foods [56]. The poor diet quality may result in insufficient intake of crucial nutrients, including vitamins, calcium, magnesium, and protein [55,57], which may be associated with the onset of metabolic abnormalities and MASLD. Furthermore, poor dietary intake and food insecurity have been related to systemic inflammation and gut microbiota dysbiosis [58,59], which are implicated in metabolic abnormalities and the pathophysiology of MASLD. Indeed, our analysis showed that individuals with food insecurity had poorer dietary quality, as assessed by the KHEI framework, than those without food insecurity. Furthermore, the association between food insecurity and MASLD was slightly attenuated after additional adjustment for the KHEI (Model 1 vs. Model 2), suggesting that dietary quality may be one factor associated with the observed association. In addition, food insecurity was associated with several metabolic abnormalities, including hypertriglyceridemia, in our additional analyses. While our analysis did not find a direct association between food insecurity and obesity or DM, previous studies using more precise measures of adiposity and insulin resistance have reported such associations. For instance, the poor dietary quality related to food insecurity may be associated with higher body fat percentage [8] and greater insulin resistance, as measured by the homeostatic model assessment of insulin resistance (HOMA-IR) [60]. Notably, one study further demonstrated that the association between food insecurity and insulin resistance was partially mediated by systemic inflammation and stress hormones, as measured by high-sensitivity C-reactive protein (hs-CRP) and cortisol [61]. These findings suggest that metabolic dysregulation related to food insecurity may be associated with the development of MASLD through pathways involving insulin resistance, chronic inflammation, and physiological stress [62]. However, the association between food insecurity and MASLD remained significant after adjusting for a range of potential confounders, including dietary quality, suggesting that additional pathways or unmeasured confounding may contribute to this association. For instance, food insecurity may serve as a marker of broader socioeconomic disadvantages, including adverse early-life socioeconomic conditions, limited social support, limited access to healthcare services, and low health literacy, which were not fully captured in the present study. Previous studies have reported that food insecurity is related to adverse childhood experiences, poor social support, and health literacy [63,64,65]. Additionally, the financial burden associated with food insecurity may limit access to healthcare services and increase medication nonadherence, which may further lead to the onset and progression of MASLD [66]. Although these mechanisms may provide plausible explanations for the observed association, these interpretations should be regarded as hypotheses rather than established mechanisms. Furthermore, given the cross-sectional observational design of the present study, the observed associations between food insecurity and MASLD should not be interpreted as causal relationships.

### Limitations

This study has several limitations. First, causal inferences regarding the relation between food insecurity and MASLD could not be drawn because of the cross-sectional design. Cohort studies should be conducted to analyze the temporal sequence between food insecurity and the subsequent onset of MASLD. Second, hepatic steatosis was determined based on the biomarker-based indices rather than imaging modalities or liver biopsy. Although the HSI and other validated indices are widely used in large-scale epidemiological studies and have demonstrated reasonable accuracy [25], they remain susceptible to misclassification errors. For instance, as noted in a previous study [25], the cutoff of an HSI >36 may result in a high false-negative rate, potentially leading to the misclassification of individuals with mild MASLD as controls. Although we employed alternative indices to assess the robustness of our findings, these measures are surrogate biomarkers rather than direct indicators of hepatic steatosis. Therefore, the association between food insecurity and MASLD may largely reflect its association with metabolic abnormalities rather than a direct association with hepatic steatosis. Therefore, the present findings should be validated in future studies using more accurate imaging-based assessments or histological confirmation. Third, food insecurity was determined using a questionnaire, which is subject to measurement error. For example, participants may have been influenced by recall or social desirability bias, potentially resulting in misclassification of food insecurity status. Fourth, because of limitations in the available data, we could not account for factors such as the use of hepatotoxic medications, family history, or genetic predisposition. Additionally, specific information on chronic illnesses and the use of multivitamins or antioxidants was not available in the dataset, which represents a limitation of this study. Consequently, residual confounding factors cannot be excluded. Fifth, as noted in a previous study [18], the association of food insecurity with MASLD may vary according to a country’s socioeconomic status and food security policies. Accordingly, the findings of this study may not extend to other socioeconomic contexts. Sixth, while the KHEI was a validated measurement for the dietary quality assessment, it is assessed based on the 24-h recall. Therefore, the KHEI might not fully capture individuals’ long-term dietary quality. Seventh, our statistical models aimed to estimate both the total association between food insecurity and MASLD and the association independent of dietary quality. Because dietary quality may represent an intermediate variable in the association between food insecurity and MASLD, the model adjusting for dietary quality should be interpreted with caution, as it may result in overadjustment when estimating the total association between food insecurity and MASLD. Eighth, some of the proposed mediating mechanisms, such as systemic inflammation, adiposity, and insulin resistance, could not be formally evaluated using mediation analyses in this study due to the lack of precise biomarkers (e.g., C-reactive protein, fat mass, or HOMA-IR) and the cross-sectional design. Future studies should therefore investigate these potential mechanisms using more comprehensive biomarker assessments.

Despite these limitations, our study makes important contributions to the literature by examining the association between food insecurity and MASLD, an understudied topic in the existing literature. Furthermore, the use of a nationally representative sample enhances the generalizability of our findings.

## 5. Conclusions

This study explored the association between food insecurity and MASLD in Korean adults. In comparison with participants with no food insecurity, those with mild and moderate-to-severe food insecurity had higher odds of MASLD. Further prospective research is needed to elucidate the temporal association between food insecurity and MASLD.

## Figures and Tables

**Table 1:** Characteristics of the study participants by food insecurity groups.

	Total	Food Insecurity Groups		
Variable		No	Mild	Moderate-to-Severe
	N = 12,697	N = 12,121	N = 474	N = 102
Sex				
Male	5457 (50.0%)	5236 (50.1%)	176 (43.4%)	45 (53.3%)
Female	7240 (50.0%)	6885 (49.9%)	298 (56.6%)	57 (46.7%)
Age				
Mean (SD)	47.4 ± 16.6	47.2 ± 16.5	52.9 ± 18.0	51.3 ± 18.6
Region				
Urban	10,122 (85.6%)	9692 (85.9%)	355 (78.1%)	75 (81.0%)
Rural	2575 (14.4%)	2429 (14.1%)	119 (21.9%)	27 (19.0%)
Income level				
Lowest	2342 (13.6%)	2024 (12.1%)	250 (48.5%)	68 (63.4%)
Low	3080 (22.6%)	2914 (22.3%)	136 (30.3%)	30 (32.2%)
High	3465 (29.4%)	3394 (30.0%)	69 (17.0%)	2 (2.5%)
Highest	3810 (34.3%)	3789 (35.5%)	19 (4.2%)	2 (1.9%)
Education level				
Middle school or below	3522 (18.9%)	3218 (18.1%)	250 (40.2%)	54 (42.8%)
High school	4270 (36.7%)	4078 (36.5%)	154 (41.1%)	38 (45.3%)
College or above	4905 (44.4%)	4825 (45.4%)	70 (18.7%)	10 (11.9%)
Marital status				
Married	8501 (63.7%)	8227 (64.4%)	248 (51.3%)	26 (25.7%)
Unmarried	4196 (36.3%)	3894 (35.6%)	226 (48.7%)	76 (74.3%)
Smoking status				
No	10,699 (81.3%)	10,252 (81.6%)	383 (77.5%)	64 (54.6%)
Yes	1998 (18.7%)	1869 (18.4%)	91 (22.5%)	38 (45.4%)
Physical activity				
No	7263 (54.2%)	6887 (53.8%)	314 (64.0%)	62 (59.1%)
Yes	5434 (45.8%)	5234 (46.2%)	160 (36.0%)	40 (40.9%)
AUDIT-C				
Mean (SD)	3.8 ± 3.6	3.8 ± 3.6	3.2 ± 3.6	4.0 ± 4.0

AUDIT-C, Alcohol Use Disorders Identification Test–Consumption; SD, standard deviation. Values are presented as unweighted raw N with survey-weighted percentages (N, %) or as survey-weighted means with standard deviations.

**Table 2:** Dietary quality across food insecurity groups, as assessed by the Korean Healthy Eating Index.

	Total	Food Insecurity Groups		
		No	Mild	Moderate-to-Severe
**Total KHEI score (range: 0 to 100)**	58.9 ± 13.3	59.0 ± 13.3	57.5 ± 13.4	50.3 ± 15.0
**Total adequacy score**	29.5 ± 10.8	29.6 ± 10.7	26.6 ± 11.1	20.5 ± 11.0
Breakfast (0–10)	6.5 ± 4.2	6.5 ± 4.2	6.7 ± 4.1	5.4 ± 4.6
Whole grains (0–5)	1.8 ± 2.1	1.8 ± 2.1	1.7 ± 2.1	1.3 ± 2.0
Total fruit (0–5)	1.9 ± 2.1	1.9 ± 2.1	1.6 ± 2.1	0.8 ± 1.6
Fruit, excluding juice (0–5)	2.1 ± 2.3	2.1 ± 2.3	1.8 ± 2.3	1.0 ± 2.0
Total vegetables (0–5)	3.4 ± 1.5	3.4 ± 1.5	3.2 ± 1.6	2.9 ± 1.6
Vegetables, excluding kimchi and pickles (0–5)	3.1 ± 1.7	3.1 ± 1.6	2.7 ± 1.7	2.5 ± 1.8
Meat, fish, eggs, and beans (0–10)	7.3 ± 3.0	7.4 ± 3.0	6.3 ± 3.3	4.9 ± 3.6
Milk and dairy (0–10)	3.3 ± 4.4	3.4 ± 4.4	2.5 ± 4.1	1.7 ± 3.7
**Total moderation score**	20.1 ± 6.3	20.0 ± 6.3	21.9 ± 6.1	22.6 ± 6.7
Saturated fatty acid (0–10)	6.6 ± 4.3	6.6 ± 4.3	7.3 ± 4.0	6.9 ± 4.3
Sodium (0–10)	7.0 ± 3.1	6.9 ± 3.2	7.6 ± 2.9	8.4 ± 2.2
Sugar (0–10)	6.5 ± 3.8	6.5 ± 3.8	7.0 ± 3.8	7.2 ± 3.6
**Total balance score**	9.3 ± 4.6	9.3 ± 4.6	9.0 ± 4.6	7.2 ± 5.1
Carbohydrate (0–5)	2.7 ± 2.1	2.7 ± 2.1	2.7 ± 2.1	2.0 ± 2.1
Fat (0–5)	3.5 ± 2.1	3.5 ± 2.1	3.5 ± 2.0	2.8 ± 2.3
Total energy (0–5)	3.1 ± 2.2	3.1 ± 2.2	2.7 ± 2.3	2.4 ± 2.2

Values are presented as weighted-adjusted means ± standard deviation.

**Table 3:** Association between food insecurity and metabolic dysfunction-associated steatotic liver disease.

	Cases/N	Model 1		Model 2	
		OR (95% CI)	*p* Value	OR (95% CI)	*p* Value
**Food insecurity groups**					
No	2894/12,121	Reference		Reference	
Mild	147/474	1.42 (1.10–1.83)	0.008	1.40 (1.09–1.81)	0.010
Moderate-to-severe	34/102	1.83 (1.10–3.02)	0.020	1.76 (1.06–2.90)	0.028
**Food insecurity score**					
HFSSM continuous scale		1.07 (1.03–1.12)	0.001	1.07 (1.02–1.12)	0.003

HFSSM, Household Food Security Survey Module; OR, odds ratio; CI, confidence interval; Model 1: sex + age + region + income + education + marital status + smoking + physical activity + alcohol use; Model 2: Model 1 + Korean Healthy Eating Index.

**Table 4:** Relationship between food insecurity and metabolic abnormalities.

	Model 1		Model 2	
Outcome Variables: Metabolic Risk Factors	OR (95% CI)	*p* Value	OR (95% CI)	*p* Value
**1. Overweight/obesity or high waist circumference**				
Food insecurity groups				
No	Reference		Reference	
Mild	1.17 (0.90–1.53)	0.249	1.16 (0.89–1.52)	0.277
Moderate-to-severe	1.09 (0.62–1.92)	0.752	1.06 (0.60–1.87)	0.834
Food insecurity score				
HFSSM continuous scale	1.02 (0.97–1.07)	0.428	1.02 (0.97–1.07)	0.518
**2. Increased glucose/HbA1c or DM mediation use**				
Food insecurity groups				
No	Reference		Reference	
Mild	1.25 (0.93–1.69)	0.138	1.24 (0.92–1.68)	0.157
Moderate-to-severe	1.01 (0.58–1.75)	0.979	0.98 (0.56–1.70)	0.932
Food insecurity score				
HFSSM continuous scale	1.02 (0.97–1.07)	0.541	1.01 (0.96–1.06)	0.643
**3. Elevated blood pressure**				
Food insecurity groups				
No	Reference		Reference	
Mild	1.05 (0.84–1.33)	0.663	1.05 (0.83–1.32)	0.687
Moderate-to-severe	1.86 (1.09–3.16)	0.023	1.84 (1.08–3.13)	0.026
Food insecurity score				
HFSSM continuous scale	1.02 (0.97–1.06)	0.522	1.01 (0.97–1.06)	0.567
**4. Hypertriglyceridemia**				
Food insecurity groups				
No	Reference		Reference	
Mild	1.18 (0.93–1.50)	0.171	1.18 (0.93–1.50)	0.178
Moderate-to-severe	1.66 (1.00–2.76)	0.050	1.65 (0.99–2.74)	0.055
Food insecurity score				
HFSSM continuous scale	1.05 (1.01–1.10)	0.020	1.05 (1.01–1.10)	0.023
**5. Reduced HDL-C**				
Food insecurity groups				
No	Reference		Reference	
Mild	1.01 (0.80–1.27)	0.941	1.00 (0.80–1.26)	0.971
Moderate-to-severe	2.14 (1.24–3.68)	0.006	2.11 (1.22–3.62)	0.007
Food insecurity score				
HFSSM continuous scale	1.05 (1.01–1.09)	0.023	1.05 (1.01–1.09)	0.027

OR, odds ratio; CI, confidence interval; SD, standard deviation; Model 1: sex + age + region + income + education + marital status + smoking + physical activity + alcohol use; Model 2: Model 1 + Korean Healthy Eating Index.

**Table 5:** Subgroup analyses on the association between the HFSSM score and MASLD.

	Model 1		Model 2	
	OR (95% CI)	*p* Value	OR (95% CI)	*p* Value
**Sex**				
Male	1.06 (0.99–1.13)	0.077	1.06 (0.99–1.13)	0.106
Female	1.09 (1.03–1.15)	0.003	1.08 (1.02–1.14)	0.006
**Age**				
<60 years	1.06 (1.00–1.12)	0.036	1.06 (1.00–1.12)	0.047
≥60 years	1.07 (1.00–1.15)	0.037	1.06 (1.00–1.14)	0.069
**Obesity**				
No	1.04 (0.94–1.15)	0.456	1.04 (0.94–1.15)	0.474
Yes	1.15 (1.06–1.24)	<0.001	1.14 (1.06–1.24)	<0.001
**Diabetes mellitus**				
No	1.04 (0.98–1.09)	0.193	1.03 (0.98–1.08)	0.272
Yes	1.08 (0.98–1.19)	0.116	1.07 (0.98–1.18)	0.139

OR, odds ratio; CI, confidence interval; Model 1: sex + age + region + income + education + marital status + smoking + physical activity + alcohol use; Model 2: Model 1 + Korean Healthy Eating Index.

## Data Availability

The dataset can be obtained at https://knhanes.kdca.go.kr (accessed on 5 May 2026).

## Supplementary Materials

The following supporting information can be downloaded at: https://www.mdpi.com/article/10.3390/nu18172806/s1, Table S1: Food security questionnaire for KNHANES; Table S2: Alternative criteria for the classification of hepatic steatosis. Table S3: Association between food insecurity and metabolic dysfunction-associated steatotic liver disease after multiple imputation (N = 14,174); Table S4: Association between food insecurity and metabolic dysfunction-associated steatotic liver disease using the FSI and ZJU index.
