# Supplementary material for: Association Between Food Insecurity and Metabolic Dysfunction-Associated Steatotic Liver Disease: A Cross-Sectional Study in South Korea
Source: Nutrients. 2026 Aug 27;18(17):2806. doi: 10.3390/nu18172806 (PMC13567249; doi:10.3390/nu18172806)
Supplement: Supplementary file 1 [file nutrients-18-02806-s001.zip › nutrients-4483701-supplementary.pdf]

Table S1. Food security questionnaire for KNHANES.

Item No.	Questionnaire	Responses	Scoring
1	During the last 12 months, in (you/others in your household) because of a lack of money for food, how often have you worried about running out food before got money to buy more?	(a) often, (b) sometimes, (c) never	(a) or (b) = 1<br>(c) = 0
2	During the last 12 months, in (you/others in your household) because of a lack of money for food, how often food just didn't last?	(a) often, (b) sometimes, (c) never	(a) or (b) = 1<br>(c) = 0
3	During the last 12 months, in (you/others in your household) because of a lack of money for food, how often you couldn't afford to eat balanced meals?	(a) often, (b) sometimes, (c) never	(a) or (b) = 1<br>(c) = 0
4	During the last 12 months, because of a lack of money for food, have (you/other adults in your household) ever cut either the size of your meals or skip meals?	(a) yes, (b) no	(a) = 1<br>(b) = 0
4-1	(if "yes" for item #4) How often?	(a) almost every month, (b) some months but not every month, (c) only one or two months	(a) or (b) = 1<br>(c) = 0
5	During the last 12 months, because of a lack of money for food, have you ever eaten less than you felt you should?	(a) yes, (b) no	(a) = 1<br>(b) = 0
6	During the last 12 months, because of a lack of money for food, have you ever not eaten although you were hungry?	(a) yes, (b) no	(a) = 1<br>(b) = 0
7	During the last 12 months, because of a lack of money for food, have you ever lost weight because you didn't eat as needed?	(a) yes, (b) no	(a) = 1<br>(b) = 0
8	During the last 12 months, because of a lack of money for food, have (you/you or other adults in your household) ever been hungry for a whole day?	(a) yes, (b) no	(a) = 1<br>(b) = 0
8-1	(if "yes" for item #8) How often?	(a) almost every month, (b) some months but not every month, (c) only one or two months	(a) or (b) = 1<br>(c) = 0
9	During the last 12 months, because of a lack of or being frugal money for food, how often have you fed your child/children with only one or two kinds of low-cost food?	(a) often, (b) sometimes, (c) never	(a) or (b) = 1<br>(c) = 0
10	During the last 12 months, because of a lack of money, how often did you not feed your child/children a balanced meal?	(a) often, (b) sometimes, (c) never	(a) or (b) = 1<br>(c) = 0
11	During the last 12 months, because of a lack of money, how often did you not feed your child/children as needed?	(a) often, (b) sometimes, (c) never	(a) or (b) = 1<br>(c) = 0
12	During the last 12 months, because of a lack of money, have you ever cut the size of your (child's/children's) meals?	(a) yes, (b) no	(a) = 1<br>(b) = 0
13	During the last 12 months, because of a lack of money, have your child/children ever skipped meals?	(a) yes, (b) no	(a) = 1<br>(b) = 0
13-1	(if "yes" for item #13) How often?	(a) almost every month, (b) some months but not every month, (c) only one or two months	(a) or (b) = 1<br>(c) = 0
14	During the last 12 months, because of a lack of money, have your child/children ever not eaten although they were hungry?	(a) yes, (b) no	(a) = 1<br>(b) = 0
15	During the last 12 months, because of a lack of money, have your child/children ever been hungry for a whole day?	(a) yes, (b) no	(a) = 1<br>(b) = 0

Table S2. Alternative criteria for the classification of hepatic steatosis.

Measures	Equation	Cutoff
FSI score[1]	−7.981 + 0.011 × age (years) − 0.146 × sex (female = 1, male = 0) + 0.173 × BMI (kg/m^2) + 0.007 × triglycerides (mg/dL) + 0.593 × hypertension (yes = 1, no = 0) + 0.789 × diabetes (yes = 1, no = 0) + 1.1 × ALT:AST ratio ≥1.33 × (yes = 1, no = 0).	> 23
ZJU index[2]	BMI (kg/m^2) + fasting glucose (mmol/L) + TG (mmol/L) + 3 × (ALT/AST) + 2 (if female)	> 38

ALT, alanine aminotransferase; AST, aspartate aminotransferase; BMI, body mass index; FSI, Framingham Steatosis Index; ZJU index, Zhejiang University index.

1 Long MT, Pedley A, Colantonio LD, Massaro JM, Hoffmann U, Muntner P, Fox CS. Development and Validation of the Framingham Steatosis Index to Identify Persons With Hepatic Steatosis. Clin Gastroenterol Hepatol. 2016 Aug;14(8):1172-1180.e2.

2 Wang J, Xu C, Xun Y, Lu Z, Shi J, Yu C, Li Y. ZJU index: a novel model for predicting nonalcoholic fatty liver disease in a Chinese population. Sci Rep. 2015 Nov 16;5:16494.

Table S3. Association between food insecurity and metabolic dysfunction-associated steatotic liver disease after multiple imputation (n = 14,174).

	Adjusted OR (95% CI)
**Food insecurity groups**	
No food insecurity	Reference
Mild food insecurity	1.39 (1.10–1.76)
Moderate-to-severe food insecurity	1.68 (1.07–2.63)
**Food insecurity score**	
HFSSM continuous scale	1.06 (1.02–1.10)

HFSSM, Household Food Security Survey Module; OR, odds ratio; CI, confidence interval.
The model was adjusted for sex, age, region, income, education, marital status, smoking, physical activity, alcohol use, and dietary quality.

Table S4. Association between food insecurity and metabolic dysfunction-associated steatotic liver disease using the FSI and ZJU index.

	FSI index	ZJU index
	**Adjusted OR (95% CI)**	**Adjusted OR (95% CI)**
**Food insecurity groups**		
No food insecurity	References	Reference
Mild food insecurity	1.36 (1.06–1.76)	1.29 (1.01–1.66)
Moderate-to-severe food insecurity	2.19 (1.31–3.68)	1.75 (1.04–2.93)
**Food insecurity score**		
HFSSM continuous scale	1.08 (1.04–1.13)	1.06 (1.02–1.11)

FSI, Framingham Steatosis Index; ZJU index, Zhejiang University index; HFSSM, Household Food Security Survey Module; OR, odds ratio; CI, confidence interval.
The models were adjusted for sex, age, region, income, education, marital status, smoking, physical activity, alcohol use, and dietary quality.
